# Indian medicinal phytocompounds for targeting apoptosis and high-penetrance genes in triple-negative breast cancer: an in-silico exploration

**DOI:** 10.1186/s12860-025-00548-6

**Published:** 2025-07-30

**Authors:** Reshmi Kumari, Satarupa Banerjee

**Affiliations:** Department of Biotechnology, School of Biosciences and Technology, VIT University Vellore, Vellore, Tamil Nadu 632014 India

**Keywords:** TNBC, Phytochemicals, Multi-targeted therapy, In-silico analysis, Natural compounds

## Abstract

Triple-negative breast cancer (TNBC) presents a significant therapeutic challenge due to its aggressive nature, lack of hormone receptors, and limited targeted treatment options. The complexity of the disease is further compounded by mutations in high-penetrance genes such as BRCA1, BRCA2, and BAX, along with other apoptotic genes involved in tumorigenesis, apoptosis, and drug resistance. Targeting these genes through innovative therapeutic approaches is crucial for improving treatment outcomes. This in-silico study explores the potential of phytochemicals as natural, multi-targeted therapeutic agents against high-penetrance and apoptotic genes implicated in TNBC. Using the IMPPAT 2.0 database, 300 phytochemicals were systematically screened based on their pharmacokinetic properties and toxicity profiles to identify promising candidates. Among them, Bayogenin exhibited strong binding to BRCA2 (-9.3 kcal/mol) and PALB2 (-8.7 kcal/mol), surpassing the FDA-approved drug Olaparib in molecular docking studies. Molecular dynamics simulations over 200 ns further confirmed the stability of these phytochemical-protein complexes, showing consistent root mean square deviation, hydrogen bonding, and free energy profiles. These findings highlight the therapeutic potential of phytochemicals and their possible advantages over existing TNBC treatments. By targeting key molecular pathways, this study provides insights into the development of natural, multi-targeted therapeutic strategies, emphasizing their translational relevance for TNBC therapy.

**Supplementary Information:**

The online version contains supplementary material available at 10.1186/s12860-025-00548-6.

## Introduction

Cancer is a group of approximately 200 different diseases, each characterized by distinct genetic, molecular, and cellular alterations that drive uncontrolled growth and spread. Breast cancer remains the most diagnosed cancer globally, with over 3.8 million new cases reported in 2023. Despite advancements in treatment, the mortality-to-incidence ratio remains high at 19.6%. Among its subtypes, TNBC poses a significant therapeutic challenge, accounting for 10–20% of all breast cancer cases [1]. TNBC is defined by the absence of estrogen receptors, progesterone receptors, and HER2 expression, making it resistant to hormone therapies and HER2-targeted treatments [2]. It predominantly affects younger women under 40 and is associated with high metastatic potential and poor prognosis. The lack of effective targeted therapies leaves chemotherapy as the primary treatment option [3]. While chemotherapy initially shows efficacy, patients often experience rapid resistance development, leading to poor survival outcomes, especially in metastatic cases. Additionally, TNBC’s heterogeneous nature complicates treatment, as different subtypes exhibit varied responses, emphasizing the need for personalized therapeutic approaches.

A promising strategy for overcoming TNBC’s therapeutic limitations involves targeting high-penetrance genes and apoptotic pathways that contribute to tumor progression, chemotherapy resistance, and poor prognosis. Genes such as BRCA1, BRCA2, BAX, and PALB2 play essential roles in DNA repair, apoptosis, and cellular homeostasis, and their mutations are frequently implicated in TNBC’s aggressive progression [4]. Targeting these genes can inhibit tumor growth, reduce metastasis, and enhance treatment response [5]. TNBC frequently exhibits dysregulated apoptotic pathways, allowing cancer cells to evade programmed cell death [6]. Restoring apoptotic regulators like BAX provides an opportunity to sensitize cancer cells to treatment and inhibit uncontrolled proliferation [7]. Given TNBC’s resistance to chemotherapy, an integrated molecular approach focusing on high-penetrance genes and apoptotic pathways holds significant therapeutic promise.

Phytochemicals have gained attention due to their ability to modulate multiple molecular targets and their potential role in cancer therapy. Notably, TNBC prevalence in India (27–31%) is significantly higher than in Western countries (12–15%) [8]. Given the genetic diversity within the Indian population, investigating phytochemicals from traditional Indian medicine could lead to personalized and culturally relevant therapeutic strategies [9]. Indian medicinal plants have been extensively used in traditional medicine and Ayurveda, making them a valuable source for therapeutic exploration. This study aims to identify plant-derived polyphenols with targeted efficacy against TNBC, particularly in populations where the disease burden is disproportionately high. This selection was based on the well-documented antioxidant and anti-inflammatory properties of polyphenols, which play a crucial role in cancer prevention and therapy. Polyphenols also exert antioxidant effects by scavenging free radicals, reducing oxidative stress, and preventing DNA damage, thereby protecting cells from carcinogenic transformations.

**Fig. 1:**
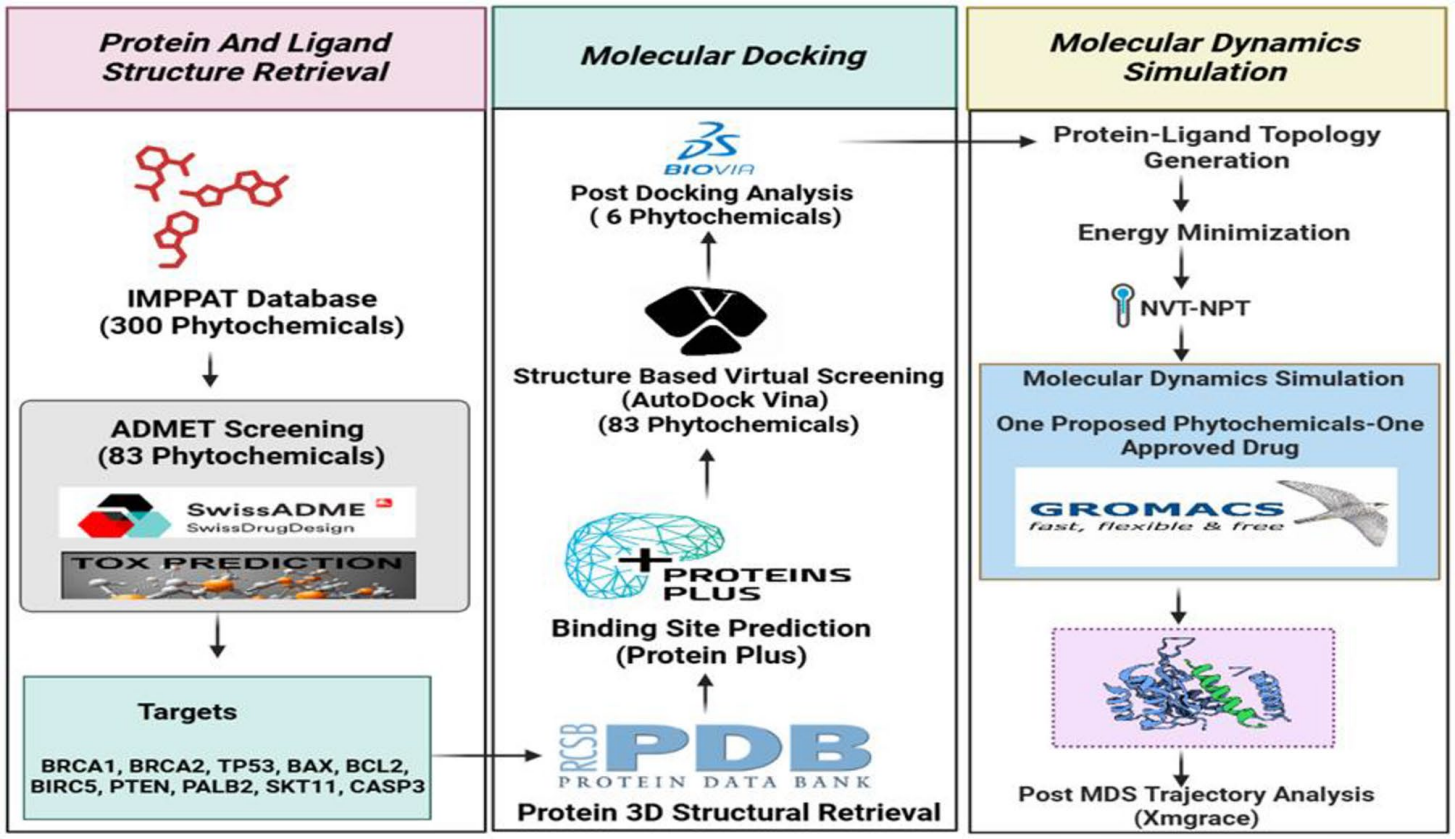
Using the IMPPAT database, 300 phytochemicals were considered as potential drug candidates against cancer-related proteins (BRCA1, TP53, etc.) retrieved from the PDB. Eighty-three of these underwent ADMET and toxicity prediction. Molecular docking (using BIOVIA and AutoDock Vina) narrowed the field to six, with binding sites predicted by Protein Plus. Molecular dynamics simulations (using GROMACS) further refined the selection to one promising phytochemical, compared against an approved drug, with final trajectory analysis performed using Xmgrace

Computer-aided drug design (CADD) is an advanced technique that involves the utilization of virtual screening, molecular docking, and molecular dynamic simulation to screen a large set of molecules to yield fewer compounds that need to be validated further. Recent research has shown the implication of these techniques in saving time, money, and manpower for drug discovery protocol [10]. By combining molecular targeting of high-penetrance genes and apoptotic pathways with phytochemical exploration, this research introduces a multi-targeted approach to TNBC treatment. This strategy enhances the potential for more effective, personalized interventions, leveraging natural compounds to address TNBC’s therapeutic challenges [11]. Through this approach, the study contributes to the growing body of research on phytochemical-based cancer therapies, offering new insights into targeted, natural treatment options for TNBC. To systematically evaluate phytochemicals for TNBC treatment, a multi-step computational approach was employed in Fig. 1.

## Materials and methods

### Ligand retrieval

The latest version, IMPPAT 2.0, comprises a comprehensive aggregation of data, encompassing 4,010 Indian medicinal flora, 17,967 phytochemicals, and 1,095 therapeutic applications. This rigorously assembled database facilitates the exploration of the interactions between plants and phytochemicals, while also advancing the identification of pharmaceuticals derived from natural products by providing valuable insights into the therapeutic properties of these botanical entities [12]. The IMPPAT database was employed to extract 300 phytochemicals following a series of meticulous screening processes. This thoughtfully organized library presents a diverse selection of polyphenolic classes, featuring flavonoids, lignans, phenolic acids, stilbenes, and non-phenolic metabolites.

The IMPAAT 2.0 database, which provides a comprehensive repository of over 4,000 Indian medicinal plants and 17,967 bioactive compounds, was selected for this study due to its well-curated and standardized data. From the vast dataset of phytochemicals in the IMPAAT database, we identified and selected polyphenols only for further analysis. Other databases containing information of medicinal plants of India, like AromaDb, NeMedPlant or BIMP were excluded since they are specific for different regions, aromatic plants, or specific bioactivity information only.

### ADMET predictions

The selected polyphenol library, consisting of 300 compounds, was subsequently analyzed using the SwissADME (Swiss absorption, distribution, metabolism, and excretion) web tool to assess compliance with the Lipinski rule of five, gastrointestinal absorption, blood-brain barrier (BBB) permeability, P-glycoprotein (PGP) substrate interaction, and the Protox-II web tool for evaluating carcinogenicity, cytotoxicity, immunotoxicity, and mutagenicity, which identified 83 phytochemicals exhibiting favorable ADMET properties and possessing potential as drug candidates [13, 14].

### Ligand Preparation

These 83 phytochemicals were sourced from the PubChem database in three-dimensional SDF file format, and the retrieved structures were prepared and minimized using the MMFF94 force field for1000 steps by means of the OpenBabel 3.3.1 tool [15].

### Selection and retrieval of protein structures

The comprehensive literature review elucidated the involvement of diverse proteins in apoptosis pathways implicated in TNBC etiology. In a parallel context, several high-penetrance genes were identified as significant regulators of TNBC [4]. The apoptotic and high-penetrance proteins were systematically identified and extracted using the Protein Data Bank (PDB) database (https://www.rcsb.org/pdb).

These proteins were selected based on their critical roles in TNBC pathogenesis, including DNA repair (BRCA1, BRCA2, PALB2), apoptosis regulation (BAX, BCL2, CASP3), and tumor suppression (PTEN, TP53, STK11). BRCA1, BRCA2, and PALB2 are essential for homologous recombination repair, and their mutations contribute to genomic instability in TNBC [16, 17]. Apoptosis regulators such as BAX, BCL2, and CASP3 play a key role in TNBC’s resistance to cell death, with BCL2 promoting survival and BAX and CASP3 facilitating programmed cell death [18, 19]. PTEN, TP53, and STK11 function as tumor suppressors, influencing pathways such as PI3K/AKT and AMPK, which are frequently dysregulated in TNBC, leading to uncontrolled proliferation and metabolic reprogramming [20, 21]. Additionally, BIRC5, a potent inhibitor of apoptosis, is often overexpressed in TNBC and contributes to therapy resistance [22, 23]. The proteins procured from the PDB for utilization in this investigation included BRCA1 (PDB ID: 1T15), BRCA2 (PDB ID: 3EU7), PALB2 (PDB ID: 2W18), PTEN (PDB ID: 1D5R), TP53 (PDB ID: 4MZI), STK11 (PDB ID: 5WXN), BCL2 (PDB ID: 6O0K), BAX (PDB ID: 2G5B), BIRC5 (PDB ID: 2QFA), and CAPS3 (PDB ID: 1NME), respectively [4, 24, 25].

### Active sites prediction

ProteinPlus integrates various functionalities, including pocket detection, ligand-binding site prediction, and annotating biologically relevant regions based on empirical data and homology modeling. The ProteinPlus webserver was employed to ascertain the active site of the proteins, which was subsequently utilized for the molecular docking of protein-ligand complexes [26].

### Protein-ligand molecular Docking

To evaluate the compound library predicated on protein-ligand binding energy, the preparation of selected ligand proteins was executed utilizing AutoDockTools version 4.2.6 [27]. In this process, protein structures were imported into the AutoDock tool, whereupon water molecules and heteroatoms present in the protein architecture were excised. Furthermore, Kollman charges and polar hydrogen atoms were integrated into the protein systems. Subsequently, the protein structures were preserved in the pdbqt format within the designated working directory. The corresponding grid boxes for the protein were generated in the subsequent phase. The grid box’s localization, spacing, and dimensions were meticulously arranged to encompass all amino acid residues within the binding pockets of the anticipated ligand through the ProteinPlus tool, as previously mentioned. Grid maps were produced by employing Autogrid within the AutoDock tool to ascertain precise grid dimensions delineated in Table S3. A self-docking procedure was performed in the experimentally proven binding pocket of the target protein to validate the docking protocol. Since all the protein targets do not have co-crystallized ligands, we validated the docking procedure with BCL2 (PDB ID: 6O0K) protein and its co-crystallized ligand (Venetoclax). This step ensured the docking protocol opted in this study to be valid and significant.

The virtual screening of the evaluated ligand library comprising 83 compounds was conducted utilizing AutoDock Vina (https://autodock.scripps.edu/) [28]. For this endeavor, the identical grid dimensions of the respective protein as previously established were employed to further scrutinize the compound library based on the docking score. An FDA-approved drug, Olaparib, was also included as a control within the same library for comparative analysis against the proposed compound(s). The virtual screening for the proteins was executed in triplicate, and the mean of the three iterations was computed to yield a significant outcome in terms of docking score, bond quantity and types, binding site, etc. The compounds exhibiting lower binding energies than the approved compounds were shortlisted for subsequent evaluation. These shortlisted compounds were then visualized using the BIOVIA Discovery Studio visualizer (https://www.3ds.com/products/biovia/discovery-studio) to identify the optimal docked pose for further assessment. The proposed compound demonstrating a superior minimum negative binding score compared to the approved compound across multiple protein targets was advanced for investigation in molecular dynamics simulation studies.

### Protein-ligand molecular dynamics simulation (MDS)

In the setting of docking studies, proteins are predominantly modeled as rigid, allowing ligands to maintain flexibility, which diminishes the potential to faithfully reproduce the dynamic characteristics intrinsic to the protein-ligand system. A comprehensive MDS analysis serves as an effective approach for elucidating the stability of the protein-ligand system, as well as identifying the principal interaction residues among amino acids that are preserved within the specific catalytic domains observable on the surface of target proteins. To further validate the stability of the selected protein-ligand system, MDS studies were conducted utilizing the GROMACS software package, version 2023.1 [29]. The topology of the respective proteins was established employing the Chaarmm27 force field and subsequently arranged within a cubic box of 1 cm, in conjunction with TIP3P water molecules. The ligand topology was constructed utilizing the SwissParam web server [30]. The overall systems were neutralized through the incorporation of sodium counter ions. This was followed by an energy minimization process for 50,000 steps, along with NVT and NPT steps for the position restraint phase, which stabilized the pressure at 1 bar and maintained the temperature at 300 K. Subsequently, an MDS simulation was executed for a duration of 200 ns to investigate the dynamic behavior of the protein-ligand system. All trajectories recorded over the 200 ns period were subsequently analyzed in terms of Root Mean Square Deviation (RMSD), Root Mean Square Fluctuation (RMSF), Radius of Gyration (RG), and Solvent Accessible Surface Area (SASA). The hydrogen bonds formed throughout the 200 ns (comprising 20,000 frames) were scrutinized utilizing Visual Molecular Dynamics (VMD) software [31]. All protein-ligand complexes associated with the proposed compounds were compared against the approved compound to identify higher or at least comparably effective hits relative to the approved drug, achieved by generating plots using the xmgrace tool. An extended MDS analysis was also done for the selected protein-ligand complexes.

### Principal component analysis (PCA) and free-energy landscape (FEL) Estimation

Principal component analysis (PCA) is utilized to elucidate the functional dynamics of biomolecules. PCA methodology was incorporated within this investigation to obtain a concise conformational space and to assess the alterations in the overall atomic movement patterns of the respective protein targets that are complexed with both the approved pharmaceutical and the investigational drug (olaparib). The eigenvectors delineate directionality, while the eigenvalues furnish the magnitude of the intensity of atomic motion. The principal components of all the protein-ligand complexes were determined via the diagonalization of the covariance matrix. Given that the initial few principal components play a critical role in representing the global dynamics of protein-ligand complexes, the first ten principal components were selected for this analysis to discern significantly collective and strongly correlated motions. Subsequently, the “gmx covar” tool within GROMACS was employed to construct and diagonalize the estimated covariance matrix, while the “gmx_anaeig” utility was utilized for the calculation of the eigenvectors associated with projection trajectories. All two-dimensional projection plots were generated utilizing the xmgrace tool. The free energy landscape (FEL) serves as a crucial metric for assessing the stability of the protein-ligand system. To accomplish this, another GROMACS utility, “gmx_sham,” was employed to generate the FELs for all protein-ligand complexes, followed by a comparative analysis of the investigational drug about the approved pharmaceutical.

### Binding free energy calculation

The selected protein-ligand complexes were further considered for the calculation of MMPBSA using gmx_MMPBSA [32]. The gmx_MMPBSA tool executed the MMPBSA script from AMBER to quantify the binding free energy through the analysis of files generated by GROMACS. The final 10 nanoseconds (1000 frames) of the molecular dynamic simulation trajectory were selected for the binding free energy calculations, as this segment exhibited stability throughout the entirety of the simulation. The binding free energy (ΔG_binding) is computed according to the following equation:

ΔG_binding = G_complex - (G_protein + G_ligand).

Where G_complex denotes the energy of the protein-ligand complex, while G_protein and G_ligand correspond to the energies of the protein and ligand, respectively.

## Results

### Ligand retrieval, ADMET prediction, and Preparation

The ligand repository consisted of 300 phytochemicals, including flavonoids, lignans, phenolic acids, stilbenes, and non-phenolic metabolites retrieved from the IMPPAT 2.0 database. All these 300 phytochemicals were passed through SwissADME (Lipinski rule of five, gastrointestinal absorption, blood-brain barrier (BBB) permeability, P-glycoprotein (PGP) substrate interaction) and the Protox-II web tool (carcinogenicity, cytotoxicity, immunotoxicity, and mutagenicity). A total of 83 phytochemicals passed the ADMET parameters. These compounds were extracted from the PubChem database in three-dimensional SDF file format. They were then processed using Open Babel GUI to generate pdbqt input files for use in AutoDock Vina. A detailed methodology has been already discussed in the methodology section.

### Protein retrieval

**Fig. 2:**
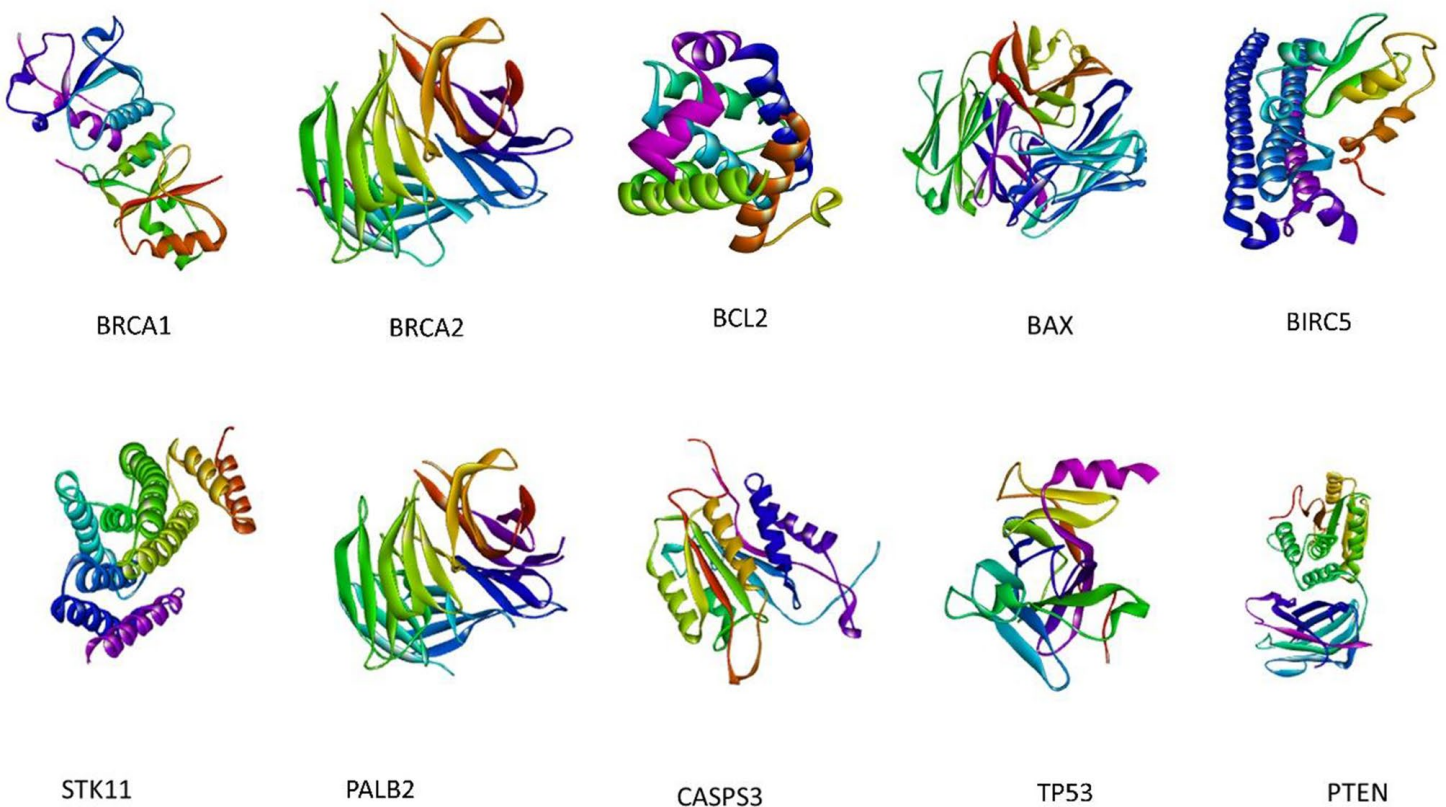
3D Structures of the crystal structure of all protein structure molecules. The figure depicts the 3D structures of several proteins implicated in cancer development and progression. Each protein is labeled accordingly

All protein molecules were meticulously chosen through an extensive review of the literature and subsequently acquired from the Protein Data Bank (PDB). The nomenclature and corresponding PDB identifiers for the selected proteins are as follows: BRCA1 (PDB ID: 1T15), BRCA2 (PDB ID: 3EU7), PALB2 (PDB ID: 2W18), PTEN (PDB ID: 1D5R), TP53 (PDB ID: 4MZI), STK11 (PDB ID: 5WXN), BCL2 (PDB ID: 6O0K), BAX (PDB ID: 2G5B), BIRC5 (PDB ID: 2QFA), and CAPS3 (PDB ID: 1NME), as illustrated in Fig. 2.

### Active site prediction and protein Preparation

All proteins examined in the present investigation were analyzed using the ProteinPlus web server, which facilitated the prediction of the binding sites for each respective residue. A comprehensive account of all proteins, including but not limited to PDB identification numbers, the resolution of the deposited structures, graphical representations, surface area, volume, and the number of residues constituting the ligand binding pocket, is provided in the supplementary material (Table S3).

### Protein-ligand molecular Docking

**Fig. 3:**
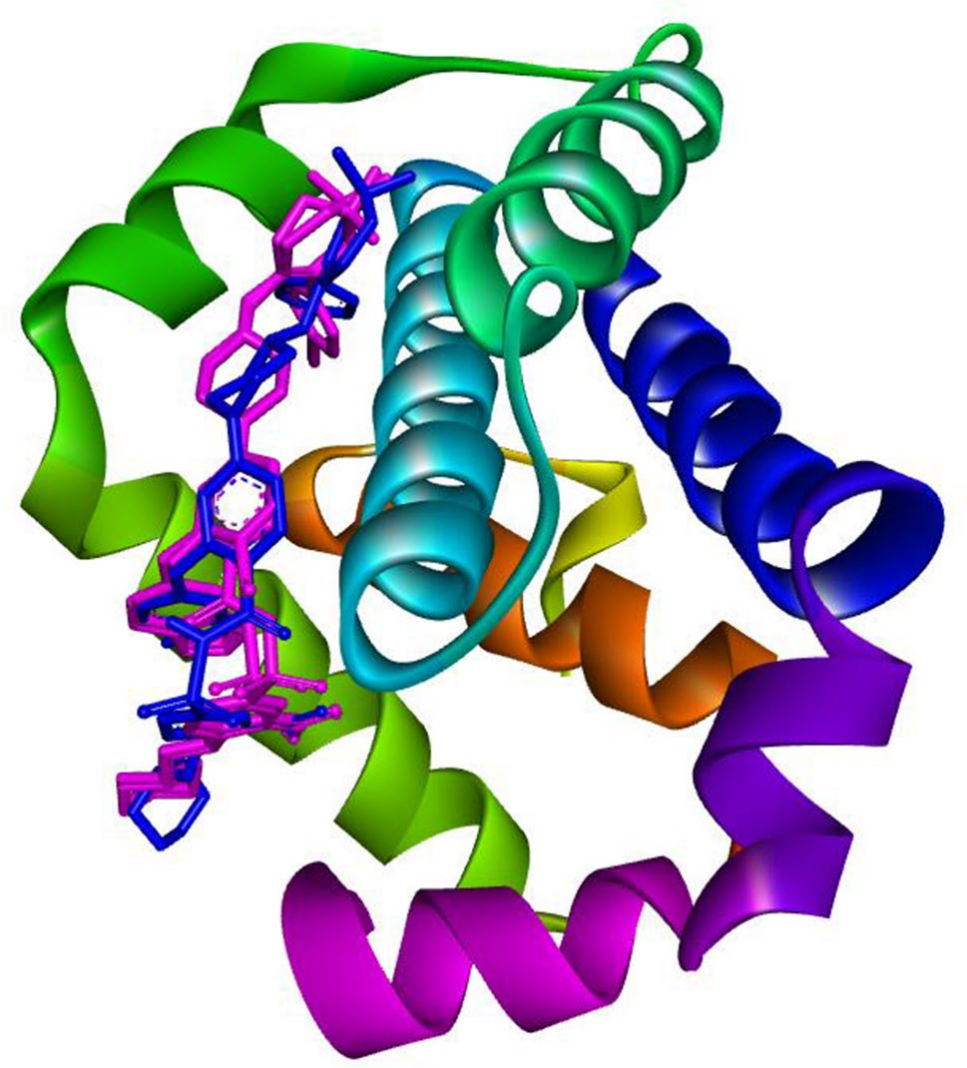
Re-docking and superimposition of co-crystallized ligand (Pink) and docked pose (Blue) in the experimentally proven binding pocket of BCL2 (PDB ID: 6O0K) protein

Protein preparation and grid generation procedures were conducted by established protocols. The co-crystallized ligand (Venetoclax) was re-docked in the experimentally proven binding pocket of BCL2 (PDB ID: 6O0K) protein to validate the docking protocol followed throughout the study. This analysis revealed the value of RMSD value of 0.72 Å upon superimposition of the co-crystallized ligand with a docked pose. The values were found within the acceptable range of RMSD i.e., < 2.0 Å. This step ensures the correct docking procedure for other protein targets as well (Fig. 3).

The dimensions of the grids (Table S3) corresponding to the individual proteins were input into the AutoDock Vina software to evaluate a ligand library composed of 83 phytochemicals alongside the FDA-approved drug Olaparib. Upon completion of the docking simulations conducted in triplicate, an average docking score was computed for each of the proposed compounds as well as for the approved compound. Subsequently, these docking scores were subjected to analysis utilizing the BIOVIA Discovery Studio visualizer. Table 1 illustrates the average docking scores for all protein targets in conjunction with the proposed ligands, which demonstrate docking scores surpassing that of the approved ligand about the respective protein. The results indicate that the proposed compounds Ajmaline, Bayogenin, Rotenone, Tephrosin, and alpha-Toxicarol exhibited superior performance compared to the approved compound (Olaparib) about docking scores assessed by the AutoDock Vina software across various receptors.

**Table 1:** Docking score (kcal/mol) of hit compounds with all the protein targets

S. no.	Compound name	BRCA1	BRCA2	BCL2	BAX	BIRC5	STK11	PALB2	CASPS3	TP53	PTEN
1	Ajmaline	-7.25	-7.8	-7.8	-6.9	-7.2	-7.5	-7.8	-6.85	-7.3	-8.2
2	Bayogenin	-7.9	-9.4	-7.9	-7.9	-8.1	-8	-9.3	-7.5	-6.6	-8.9
3	Rotenone	-7.9	-8.1	-9.1	-8.2	-7.1	-7.8	-6.0	-7.2	-7.4	-7.9
4	Sym-homospermidine	-4	-4.3	-4.7	-3.7	-4.45	-3.8	-4.2	-4.15	-3.7	-3.9
5	Tephrosin	-7.55	-8.8	-8.9	-7.7	-7.45	-7.9	-8.9	-7.75	-7.3	-7.9
6	alpha-Toxicarol	-8.1	-9.1	-7.9	-8	-7.5	-8.7	-8.6	-7.45	-7.2	-8.2
**7**	**Olaparib**	**-8.5**	**-9.4**	**-9**	**-7.6**	**-8.4**	**-8.9**	**-7.7**	**-8.9**	**-7.7**	**-9.3**

Binding energy is a critical parameter in molecular docking studies, as it quantifies the strength and stability of interactions between a ligand and a target protein. Lower binding energies indicate stronger and more stable interactions, which are often associated with higher binding affinity. In a therapeutic context, this suggests that the phytochemical has the potential to effectively engage with its target proteins, enhancing its efficacy as a drug candidate.

A comprehensive evaluation of the docking scores revealed that Bayogenin achieved scores of -9.4 kcal/mol, -7.9 kcal/mol, and − 9.3 kcal/mol, while the approved drug Olaparib registered scores of -9.4 kcal/mol, -7.6 kcal/mol, and − 7.7 kcal/mol against the three protein targets BRCA2, BAX, and PALB2, respectively. The proposed compounds Rotenone, Tephrosin, and alpha-toxicarol exhibited higher docking scores than the approved compounds across two protein targets. The docking score for Rotenone was recorded at -9.1 kcal/mol and − 8.2 kcal/mol, whereas Olaparib displayed scores of -9 kcal/mol and − 7.6 kcal/mol for the protein targets BCL2 and BAX, respectively. The docking score for Tephrosin was noted as -7.7 kcal/mol and − 8.9 kcal/mol, while Olaparib presented scores of -7.6 kcal/mol and − 7.7 kcal/mol for the protein targets BAX and PALB2, respectively. Likewise, alpha-Toxicarol yielded docking scores of -8 kcal/mol and − 8.6 kcal/mol, whereas Olaparib was measured at -7.6 kcal/mol and − 7.7 kcal/mol for the protein targets BAX and PALB2, respectively. Another noteworthy compound, Ajmaline, obtained a docking score of -7.8 kcal/mol, whereas Olaparib recorded a score of -7.7 kcal/mol for the protein target PALB2.

**Fig. 4:**
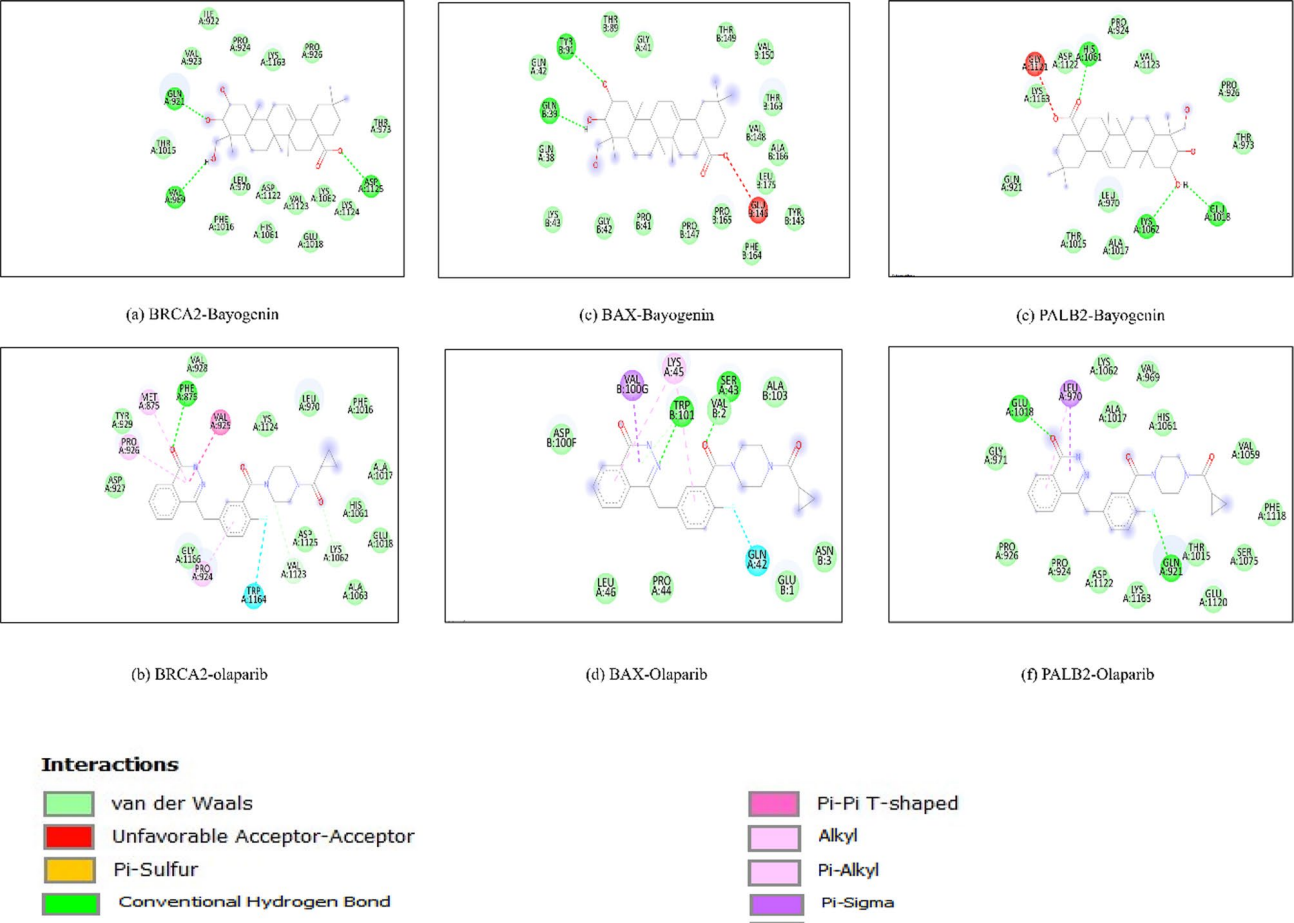
Schematic 2D representation of binding residue configuration in the docked protein-ligand complexes (**a**) BRCA2-Bayogenin, (**b**)BRCA2-Olaparib, (**c**)BAX-Bayogenin, (**d**) BAX-Olaparib, (**e**) PALB2-Bayogenin and (**f**) PALB2-Olaparib

Furthermore, through the examination of docked poses utilizing the Discovery Studio analyzer, it was determined that Bayogenin exhibited a higher frequency of conventional hydrogen bonds in comparison to the approved compound across multiple sites. The BRCA2-Bayogenin complex demonstrated the formation of three conventional hydrogen bonds involving the amino acid residues Gln921, Val969, and Asp1125. Conversely, the BAX-Olaparib complex established merely one conventional hydrogen bond with the Phe876 amino acid residue, which facilitated Bayogenin’s superiority over Olaparib. The BAX-Bayogenin complex manifested two conventional hydrogen bonds with the residues Gln39 and Tyr91, while the BAX-Olaparib complex presented two conventional hydrogen bonds at the residues Ser43 and Trp101. Furthermore, additional interaction modalities were also identified, which rendered the BAX-Olaparib complex more favorable at this juncture compared to the BAX-Bayogenin complex. The PALB2-Bayogenin complex displayed three conventional hydrogen bonds at Glu1018, His1061, and Lys1062, in contrast to the PALB2-Olaparib complex, which exhibited two conventional hydrogen bonds at the residues Gln921 and Glu1018, thereby again establishing the superiority of the PALB2-Bayogenin complex over the PALB2-Olaparib complex. Other significant interactions within the respective protein-ligand complexes have been delineated in (Table 2). A detailed description of the ADMET predictions of the filtered compounded has been shown in Table S1 and Table S2. Furthermore, representations of both 2D interactions among the respective protein-ligand complexes are illustrated in Fig. 4.

**Table 2:** Molecular interactions of BRCA-2, BAX, and PALB-2 with Bayogenin and Olaparib

S.No.	Protein-ligand complexes	Docking score (kcal/mol)	Conventional H-bond and Carbon hydrogen bond	Other interactions
1.	BRCA2-Bayogenin	-9.4	THR 973, ASP 1125	VD: ALA 922, VAL 923, PRO 924, PRO 926, LYS 1163, ASN 973, LEU 1070, ASP 1010, LEU 1015, PHE 1016, HIS 1061, GLU 1018, THR 1020, VAL 1123, LYS 1062, LYS 1124
2.	BRCA2-Olaparib	-9.4	ASP 1125, PRO 926	VD: VAL 928, VAL 925, LYS 1124, LEU 970, PHE 1016, ALA 1017, HIS 1061, GLU 1018, ALA 1063, ASP 1125, VAL 1123, LYS 1062, LEU 1070, PHE 876, TYR 929, TRP 1164, ALA 927, GLY 1166 Pi: TYR 929, PHE 876
3.	BAX-Bayogenin	-7.9	TYR:91, THR:89, THR:149	VD: GLN:38, GLN:39, GLN:42, LYS:43, GLY:41, GLY:42, PRO:147, PRO:165, VAL:148, VAL:150, ALA:166, TYR:143, PHE:164, LEU:175 UAA: GLU:146
4.	BAX-Olaparib	-7.6	GLN:42, SER:43	VD: ASP:100, VAL:100, ALA:103, LEU:46, PRO:44, ASN:3, GLU:1 HAB: GLN:42 Pi: TRP:101, VAL:2, LYS:45, VAL:100G
5.	PALB2-Bayogenin	-9.3	HIS:1061, ASP:1122, GLU:1018, LYS:1062	VD: GLN:921, PRO:924, PRO:926, VAL:1123, ALA:1017, THR:1015, LEU:970, UAA: GLY:1121, LYS:1163
6.	PALB2-Olaparib	-7.7	GLN:921, GLU: 1018	VD: PRO:924, PRO:926, VAL969, GLY:971, THR:1015, ALA:1017, GLU:1018, VAL:1059, HIS1061, LYS: 1062, SER:1075, PHE:1118, GLU:1120, LYS:1163 Pi: LEU: 970

Upon evaluating the hit compounds, it was discerned that Bayogenin demonstrated relatively superior performance compared to the other hit compounds, as evidenced by its higher or nearly equivalent docking scores about the approved drug. Bayogenin excelled across three targets, while the other hits were effective on only two or one targets, as elaborated previously. The quantity of conventional hydrogen bonds further substantiated Bayogenin advantage over the approved compound. Given the intense competition between the shortlisted drug (Bayogenin) and the approved drug (Olaparib), there arose a necessity for additional analysis, which was subsequently conducted through MDS.

### Molecular dynamics simulation (MDS) studies

Molecular Dynamics Simulation (MDS) simulations provide insights into the stability of these protein-ligand complexes under physiological conditions. A stable interaction over time suggests that the compound can maintain its binding to the target protein despite environmental fluctuations. Proteins within a solution milieu exhibit stochastic migration across a designated temporal spectrum while displaying pronounced oscillatory behaviors. MDS provides a more reliable assessment of the stability of protein-ligand complexes compared to traditional molecular docking approaches. A time-resolved MDS was performed for a duration of 200 ns concerning six protein-ligand complexes delineated during the preliminary virtual screening phases, specifically BRCA2-Bayogenin, BRCA2-Olaparib, BAX-Bayogenin, BAX-Olaparib, PALB2-Bayogenin, and PALB2-Olaparib, utilizing Gromacs-23.1 software.

#### RMSD trajectory analysis

**Fig. 5:**
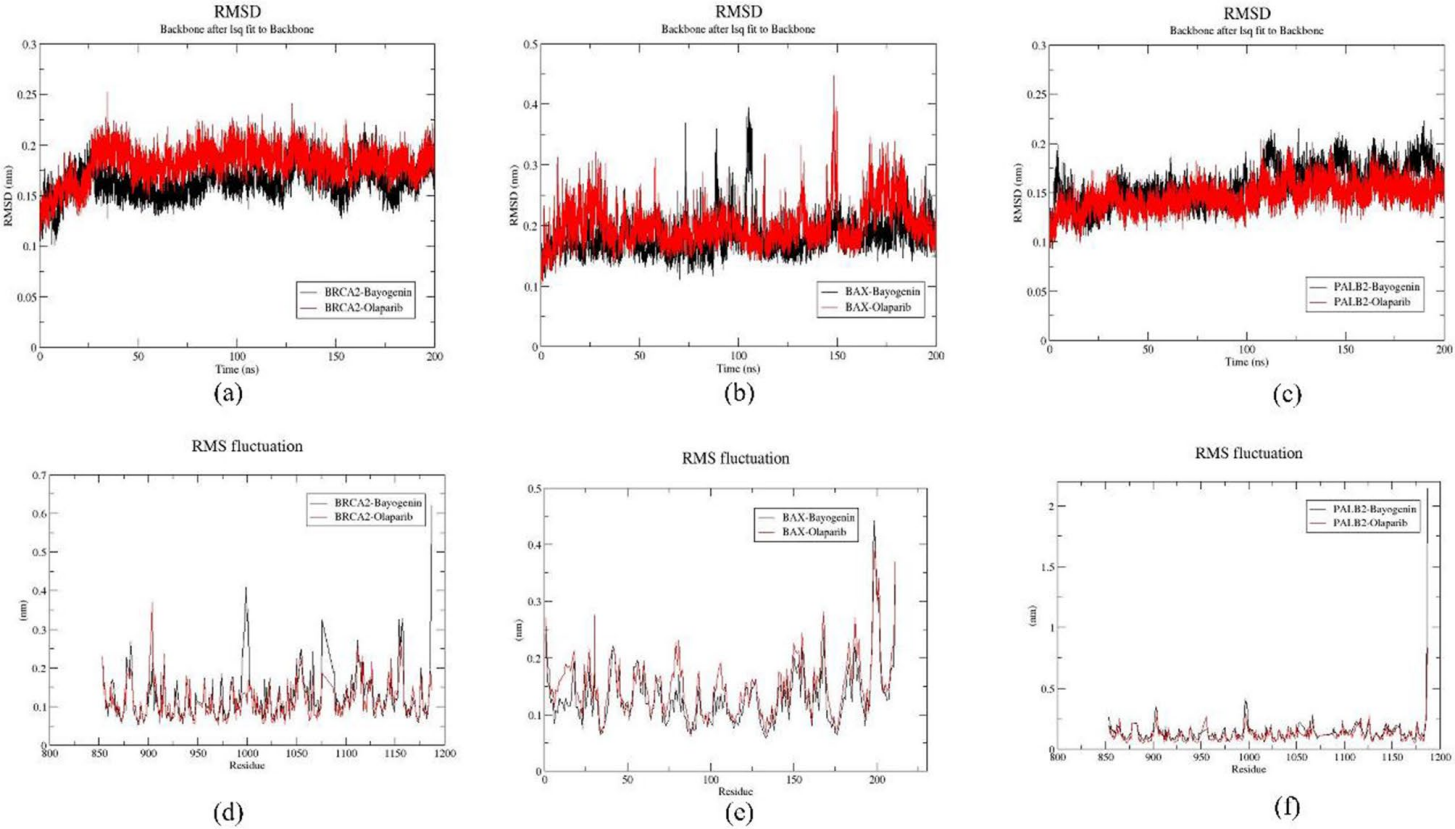
Representation of RMSD plot for (**a**) BRCA2-Bayogenin (black) and BRCA2-Olaparib (red), (**b**) BAX-Bayogenin (black) and BAX-Olaparib (red), (**c**)PALB2-Bayogenin (black) and PALB2-Olaparib (red), and representation of RMSF plot for (**d**) BRCA2-Bayogenin (black) and BRCA2-Olaparib (red), (**e**) BAX-Bayogenin (black) and BAX-Olaparib (red), (**f**) PALB2-Bayogenin (black) and PALB2-Olaparib (red)

The Root Mean Square Deviation (RMSD) plot provides a quantitative assessment of the extent of structural deviation exhibited by an atomic group relative to its original reference structure, as it evolves to its final conformation throughout the simulation. Generally, diminished RMSD values are indicative of enhanced stability, while elevated values suggest a decrease in stability. To scrutinize the stability of six protein-ligand complexes and elucidate the behavior of these systems, the RMSD values were meticulously evaluated over time. The accompanying Fig. 5. illustrates the RMSD plots corresponding to all six protein-ligand complexes identified in the preceding analysis. Concerning the protein target BRAC2, both Bayogenin and Olaparib achieved equilibrium at 25 ns, after which both plots exhibited stability. Minor fluctuations were recorded at specific time intervals of 85.4 ns, 108 ns, 130 ns, and 167 ns within the BRAC2-Bayogenin complex, whereas fluctuations at 127 ns and 115 ns were observed in the BRAC2-Olaparib complex, respectively. Both complexes demonstrated RMSD values within the range of (0.1–0.25) nm, which is within acceptable parameters. Furthermore, the average RMSD values for the BRAC2-Bayogenin and BRAC2-Olaparib complexes were determined to be 0.1466 ± 0.015569 nm and 0.1489 ± 0.016892 nm, respectively, throughout the 200 ns simulation period (Fig. 5a.). For the additional protein target BAX, the BAX-Bayogenin complex reached equilibrium at 8 ns, while the BAX-Olaparib complex attained equilibrium at 30 ns, with significant fluctuations discernible in both RMSD plots (Fig. 5b.). Notably, the primary fluctuations in the BAX-Bayogenin complex were recorded at 73 ns, 89 ns, and 107 ns, whereas in the BAX-Olaparib complex, they were noted at 148 ns. Moreover, two minor fluctuations were observed in the BAX-Olaparib complex at 112 ns and 123 ns. The average RMSD values were calculated to be 0.1654 ± 0.031052 nm and 0.1863 ± 0.035321 nm for the BAX-Bayogenin and BAX-Olaparib complexes, respectively. Overall, the RMSD trajectory of the BAX-Bayogenin complex exhibited comparatively greater stability than that of the BAX-Olaparib complex over the 200 ns period. The range of RMSD values was found to be (0.1–0.3) nm for the BAX-Bayogenin complex and (0.1–0.4) nm for the BAX-Olaparib complex. In the context of the PALB2 protein target, both the PALB2-Bayogenin complex and the PALB2-Olaparib complex reached equilibration at 3 ns. The PALB2-Bayogenin complex presented minor fluctuations at 112 ns, 145 ns, 164 ns, and 188 ns, while in the PALB2-Olaparib complex, fluctuations were observed at 108 ns and 122 ns. The range of RMSD values was determined to be (0.1–0.2) nm for both complexes, with average RMSD values recorded at 0.1377 ± 0.019289 nm and 0.1277 nm for the PALB2-Bayogenin and PALB2-Olaparib complexes, respectively (Fig. 5c.). A comparative analysis of the RMSD plots for all six protein-ligand complexes reveals that both Bayogenin and Olaparib exhibited relative stability in the context of RMSD evaluation.

#### RMSF trajectory analysis

The Root Mean Square Fluctuation (RSMF), representing the average structural deviation over time for each protein residue commencing from its initial reference position, was computed to evaluate the backbone stability of all six protein-ligand complexes during the simulation. Generally, diminished RMSF values are indicative of reduced individual flexibilities of each residue and enhanced stability of the protein-ligand complex. For the protein target BRAC2, the residues analyzed ranged from 850 to 1186. Both the BRAC2-Bayogenin and BRAC2-Olaparib complexes exhibited analogous patterns of fluctuating residues. Notably, residues 905, 998, 1079, 1090, and 1156 emerged as the principal fluctuating residues. The average RMSF values were determined to be 0.1277 ± 0.063926 nm and 0.1151 ± 0.046421 nm for the BRAC2-Bayogenin and BRAC2-Olaparib complexes, respectively (Fig. 5d.). In the case of the BAX-Bayogenin and BAX-Olaparib complexes, the residues included in the analysis spanned from 1 to 210, as per the crystal structure of the BAX protein. Numerous fluctuating residues were identified within this protein in association with both Bayogenin and Olaparib ligand molecules, which indicated comparatively lower stability in both the BAX-Bayogenin and BAX-Olaparib complexes relative to the other target protein. Average RMSF values of 0.1330 ± 0.054297 nm and 0.1532 ± 0.055607 nm underscored the relative stability of the BAX-Bayogenin complex over the BAX-Olaparib complex (Fig. 5e). Among the other protein-ligand complexes, both Bayogenin and Olaparib displayed reduced fluctuation in the RMSF plot for the protein target PALB2. The residues deemed relevant for this study were those ranging from 850 to 1185 according to the crystal structure of the PALB2 protein. Minor fluctuations were observed at residues 901 and 998 in both the PALB2-Bayogenin and PALB2-Olaparib complexes, displaying similar patterns and average RMSF values of 0.1431 ± 0.126726 nm and 0.1181 ± 0.062665 nm, respectively (Fig. 5f.). A comparative analysis of the RMSF plots across all six protein-ligand complexes reveals that both Bayogenin and Olaparib exhibited analogous patterns of fluctuations and stability across all three proteins.

#### Rg trajectory analysis

**Fig. 6:**
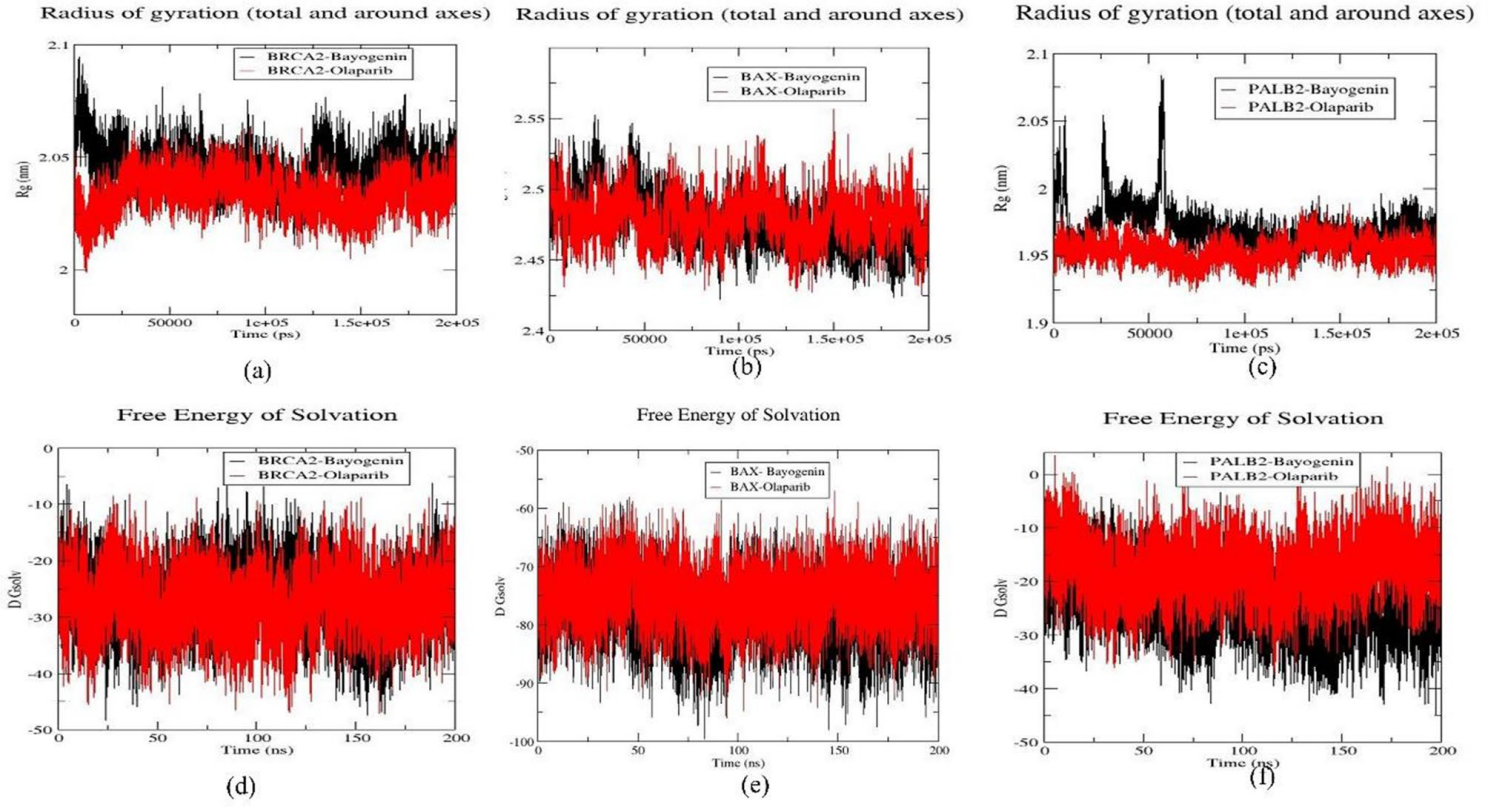
Representation of Rg plot for (**a**)BRCA2-Bayogenin (black) and BRCA2-Olaparib (red), (**b**) BAX-Bayogenin (black) and BAX-Olaparib (red), (**c**)PALB2-Bayogenin (black) and PALB2-Olaparib (red), and representation of SASA plot for (**d**)BRCA2-Bayogenin (black) and BRCA2-Olaparib (red), (**e**) BAX-Bayogenin (black) and BAX-Olaparib (red), (**f**) PALB2-Bayogenin (black) and PALB2-Olaparib (red)

The radius of gyration (Rg) quantifies the extent of compactness within the protein-ligand complex. Generally, diminished Rg values are indicative of enhanced compactness and tighter folding of the protein structure. In the present investigation, Rg was computed to assess the impact of drug binding on the compactness of the corresponding proteins. Throughout the simulation for the BRCA2-Bayogenin and BRCA2-Olaparib complexes, Rg values were observed to fall within the range of (2-2.1) nm, with average Rg values calculated at 2.0687 ± 0.064942 nm and 2.0998 ± 0.008567 nm, respectively. For the BAX-Bayogenin and BAX-Olaparib complexes, Rg values ranged from (2.42–2.55) nm, with average values determined at 2.4761 ± 0.017794 nm and 2.4799 ± 0.016624 nm throughout the 200 ns trajectories. Similarly, the Rg range for the PALB2-Bayogenin and PALB2-Olaparib complexes was identified within (1.93–2.055) nm, yielding average Rg values of 1.9712 ± 0.016055 nm and 1.9537 ± 0.008912 nm, respectively. A comparative analysis of the Rg profiles for Bayogenin and Olaparib across the three protein targets revealed that both compounds exhibited comparable compactness concerning the BAX protein, thereby indicating greater stability of the BAX-Bayogenin complex in contrast to the other complexes (Fig. 6).

#### SASA trajectory analysis

SASA quantifies the accessible surface area of proteins that can interact with solvent molecules. In this investigation, the SASA values of all six protein-ligand complexes were computed and scrutinized over a simulation duration of 200 ns. The trend observed across all six protein-ligand systems was remarkably similar; however, their mean SASA values exhibited slight variations. The calculated average SASA values were determined to be -24.95 ± 5.579925 nm², 27.75 ± 5.397654 nm², -75.87 ± 5.196412 nm², -74.87 ± 4.765381 nm², -19.86 ± 5.631884 nm², and − 13.75 ± 5.166198 nm² for the complexes BRCA2-Bayogenin, BRCA2-Olaparib, BAX-Bayogenin, BAX-Olaparib, PALB2-Bayogenin, and PALB2-Olaparib, respectively. Upon comparison of the six protein-ligand complexes, it is apparent that the average SASA value for Bayogenin in the BRCA2 complex was lower than that for Olaparib, indicating greater stability of the BRCA2-Bayogenin complex relative to the BRCA2-Olaparib complex in (Fig. 6.).

#### H-Bond analysis

**Fig. 7:**
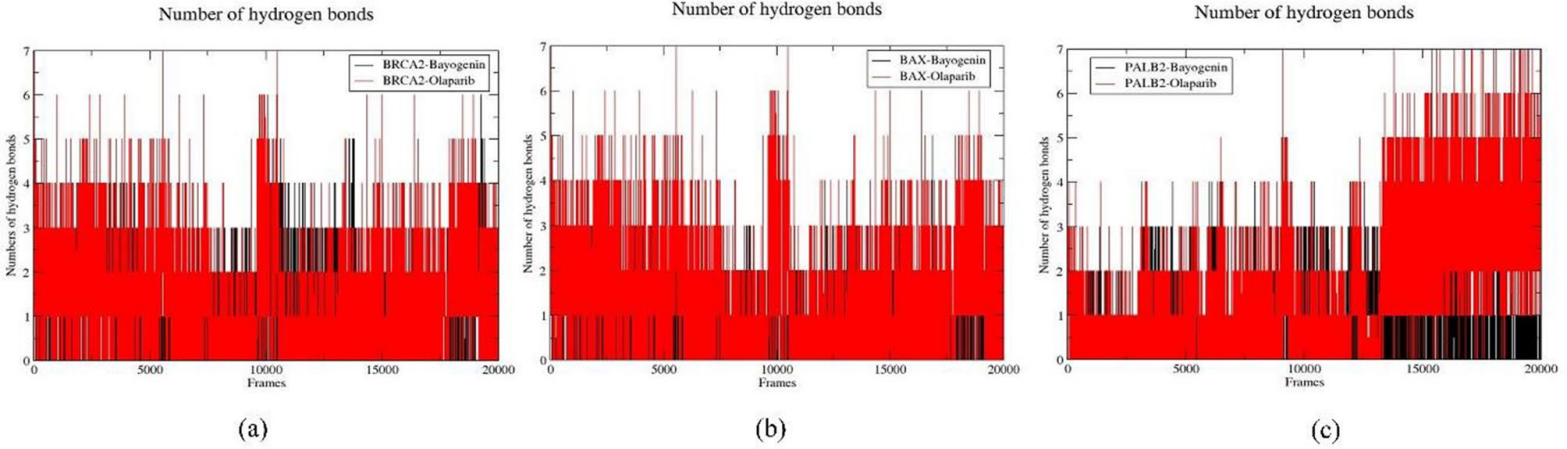
Representation of H-bond and PCA, (**a**) BRCA2-Bayogenin (black) and BRCA2-Olaparib (red), (**b**) BAX-Bayogenin (black) and BAX-Olaparib (red), (**c**) PALB2-Bayogenin (black) and PALB2-Olaparib (red)

Hydrogen bonding within a protein-ligand complex constitutes a fundamental determinant contributing to the stability of said complex. An increased quantity of hydrogen bonds correlates positively with enhanced stability of the protein-ligand complex. In the present investigation, the cumulative number of hydrogen bonds throughout 200 nanoseconds of simulation was systematically evaluated across all six protein-ligand systems. Within the BRCA2-Bayogenin and BRCA2-Olaparib complexes, three and four hydrogen bonds, respectively, were recorded throughout the simulation interval. Similarly, in the BAX-Bayogenin and BAX-Olaparib complexes, two and three hydrogen bonds were documented during the simulation period. Likewise, in the PALB2-Bayogenin and PALB2-Olaparib complexes, three and four hydrogen bonds were predominantly observed. Notably, it is important to highlight that across all three protein targets, Bayogenin exhibited the highest count of hydrogen bonds, ranging from four to six, while Olaparib presented a maximum of seven hydrogen bonds (Fig. 7.). From the analysis of hydrogen bonds, it becomes evident that while Bayogenin does not surpass Olaparib in total hydrogen bond formation, the relatively minor differences observed with protein targets such as BRCA2 underscore the significance of Bayogenin with the protein targets BRCA2, BAX, and PALB2.

#### Principal component analysis (PCA) and free energy landscape (FEL)

All six protein-ligand complexes underwent a thorough examination utilizing Principal Component Analysis (PCA) and Free Energy Landscape (FEL) plots. The PCA plots facilitate the estimation of the overall fluctuations of the Cα atom, which enables the diagonalization of covariance matrices. A comprehensive analysis of the 200 ns trajectory was conducted for Bayogenin and Olaparib across all three protein targets, namely BRCA2, BAX, and PALB2. The accompanying (Fig. S1.). illustrates the PCA plot for all six protein-ligand systems. Within a PCA plot, a reduction in conformational space correlates with diminished overall motion of the protein, thereby indicating enhanced stability of the protein-ligand system. Olaparib exhibited a reduced conformational space across all three protein targets; however, Bayogenin demonstrated a comparable conformational space to Olaparib specifically on the BAX target, thereby underscoring the significance of Bayogenin for the BAX protein as deduced from the PCA analysis.

In addition to PCA, the FEL plot serves as a pertinent parameter for the assessment of the stability of the protein-ligand system. In the context of a FEL plot, singular low-energy basins (depicted as deep blue or dark regions) are indicative of energetically favorable conformations and signify a higher stability of the protein-ligand system. As observed in (Fig. S2), Bayogenin on BRCA2 exhibited comparatively narrower low-energy basins relative to Olaparib, thereby highlighting the superior stability of Bayogenin over Olaparib concerning the BRCA2 target. Both Bayogenin and Olaparib yielded comparable results concerning low-energy basins and stability on PALB2. Conversely, in the case of the BAX protein, Olaparib was found to exhibit greater stability than Bayogenin.

**Fig. 8:**
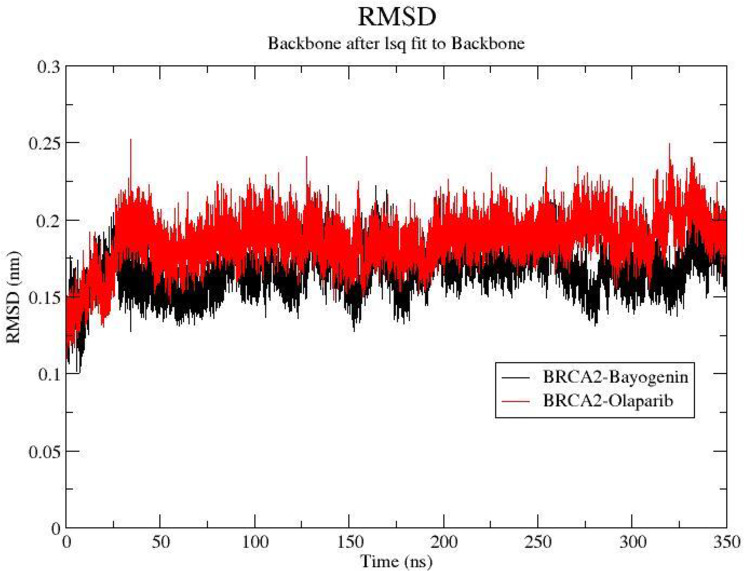
Representation of RMSF plot of BRCA2-Bayogenin (black) and BRCA2-Olaparib (red) for extended 350 ns MDS

In summary, after comparing all the plots such as RMSD, RMSF, Rg, SASA, H-bond analysis for BRCA2-Bayogenin, BRCA2-Olaparib, BAX-Bayogenin, BAX-Olaparib, PALB2-Bayogenin, and PALB2-Olaparib, it was clear that both Bayogenin and Olaparib performed better on BRCA2 target than the BAX and PALB2 target. To further confirm the stability of the protein-ligand complex of Bayogenin and Olaparib on the BRCA2 targets we extended the simulation to 350 ns for more reliable and significant results. The outcome of the extended MDS revealed that Bayogenin performed better than Olaparib on the BRCA2 protein target (Fig. 8.)

### MMPBSA

MMPBSA was performed for the protein-ligand complex BRCA2-Bayogenin and BRCA2-Olaparib to further analyze the stability using the gmx_MMPBSA tool. In general, more negative is the total free binding energy of a protein-ligand complex, the more is its stability. The outcome from the MMPBSA showed that Bayogenin exhibited higher negative binding energy (-31.59 ± 5.47 kcal/mol) than Olaparib (-25.82 ± 3.09 kcal/mol) on BRAC2 protein. The total free binding energy revealed higher stability of Bayogenin than Olaparib. Table 3 shows detailed information about different forms of binding energy.

**Table 3:** MMPBSA calculations, where: ΔVDWAALS: Van der Waals contribution, ΔEEL: electrostatic energy, ΔEGB: Polar solvation free energy, ΔESURF: nonpolar solvation free energy, ΔGGAS: gas face free energy, ΔGSOLV: solvation energy, ΔTOTAL: total calculated free binding energy i.e. ΔGBinding (all units are reported in kcal/mol)

Energy Component (kcal/mol)	BRCA2-Bayogenin	BRCA2-Olaparib
ΔVDWAALS	-30.16 ± 3.21	-41.27 ± 2.3
ΔEEL	-15.31 ± 10.5	-17.79 ± 6.28
ΔEGB	18.21 ± 5.61	38.5 ± 4.7
ΔESURF	-4.32 ± 0.36	-5.27 ± 0.27
ΔGGAS	-45.48 ± 10.7	-59.06 ± 6.7
ΔGSOLV	13.89 ± 5.41	33.24 ± 4.54
ΔTOTAL	-31.59 ± 5.47	-25.82 ± 3.09

## Discussion

This investigation highlights the therapeutic potential of phytochemicals, particularly Bayogenin, in targeting critical high-penetrance genes and apoptotic pathways implicated in TNBC. Through in-silico analyses, Bayogenin demonstrated considerable binding affinity and stability toward key TNBC-related targets, including BRCA2, BAX, and PALB2, positioning it as a promising natural, multi-targeted therapeutic agent [33, 34]. Molecular docking assessments revealed Bayogenin’s strong interactions with the WD40 domain of PALB2, characterized by significant hydrogen bonding and a binding energy of -9.3 kcal/mol. The stability of these protein-ligand complexes is vital for modulating molecular pathways involved in TNBC progression. In comparison, Bayogenin exhibited binding affinity and interaction stability comparable to, or superior to, Olaparib, a clinically approved PARP inhibitor, underscoring its therapeutic viability in TNBC treatment. The unique interactions observed for Bayogenin suggest that it may modulate PARP activity through a complementary or partial inhibition mechanism, rather than functioning as a direct competitive inhibitor like Olaparib. Additionally, Bayogenin’s structure-activity relationship is primarily influenced by its steroidal backbone, hydroxyl groups, and carbonyl groups. The hydroxyl groups facilitate hydrogen bonding with protein targets, enhancing binding affinity. The steroidal backbone enables Bayogenin to fit into specific binding pockets, while the carbonyl group allows for interactions with nucleophilic residues in protein active sites.

RMSD analyses demonstrated Bayogenin’s stability in protein-ligand complexes, particularly with BRCA2 and BAX, as it maintained lower RMSD values during 200 ns simulations, signifying structural stability. The Radius of Gyration (Rg) graphs illustrated Bayogenin’s capacity to maintain protein compactness, further validating its stable interactions with target proteins. RMSF analyses showed consistent variations across critical residues, indicating Bayogenin’s adaptability and efficacy in binding active sites. Notably, Bayogenin displayed lower average RMSF values compared to Olaparib, particularly within the PALB2 complex, highlighting its role in stabilizing critical protein regions involved in DNA repair and tumor suppression.

FEL plots revealed narrow and deep low-energy basins for the Bayogenin-protein complexes, especially with BRCA2, denoting energetically favorable conformations. This indicates that Bayogenin forms highly stable interactions within the binding pockets of its target proteins. Also, hydrogen bonding profiles showed a higher frequency of conventional hydrogen bonds in Bayogenin-protein complexes, further substantiating its stable and effective binding mechanisms.

Since Olaparib is a known PARP inhibitor with a clear mechanism of action, we docked both Bayogenin and Olaparib into the PARP protein’s binding site. This analysis helps us understand whether Bayogenin acts in the same way as Olaparib or if it uses a different approach. Molecular docking was performed using the PARP target protein (PDB ID: 7KK4), and the binding affinities of both Bayogenin and Olaparib were assessed. The docking results showed that Bayogenin exhibited a docking score of -10.3 kcal/mol, while Olaparib demonstrated a slightly stronger binding affinity with a score of -11.1 kcal/mol.

Bayogenin was found to interact with GLN759, ASP766, ASN767, LEU769, ASP770, HIS862, GLY863, SER864, ARG865, ASN868, ILE872, TYR889, MET890, TRY896, TYR907, and GLU988, whereas Olaparib interacted with ASP766, LEU769, TRP861, HIS862, GLY863, ARG878, ILE879, ALA880, PRO881, TYR889, GLY894, ILE895, TYR896, PHE897, ALA898, LYS903, SER904, TYR907, TYR910, and GLU988 residues. The differences in uncommon binding interactions suggest that Bayogenin may engage with PARP protein in a manner distinct from Olaparib, potentially influencing its inhibitory mechanism. The slightly lower binding affinity of Bayogenin suggests it may inhibit PARP through a complementary or partial mechanism, rather than acting as a direct competitive inhibitor like Olaparib.

Our findings align with previous studies that highlight Bayogenin’s potential as an anticancer compound, particularly in hormone receptor-positive breast cancer cells like MCF-7 [35, 36]. Molecular docking analysis revealed that Bayogenin binds to the progesterone receptor with a binding energy of -6.9 kcal/mol, suggesting its role in modulating breast cancer pathways. Its high bioactivity score toward nuclear receptors is consistent with its proven capability to inhibit cancer cell proliferation. Moreover, Bayogenin is reported to possess anti-inflammatory, antimicrobial, and antioxidant properties, which may contribute to its diverse therapeutic roles in TNBC management [37]. This study also emphasizes the relevance of phytochemicals in addressing TNBC heterogeneity, particularly within the Indian demographic, where TNBC prevalence is significantly higher [8].

Additionally, Bayogenin showed a strong interaction with Hexokinase 2 (HK2), a key enzyme involved in cancer cell metabolism. The docking study demonstrated a stable binding affinity (-7.2 kcal/mol), and molecular dynamics simulations further confirmed this stability. The Bayogenin-HK2 complex had a lower root mean square deviation (RMSD) value (0.79 nm) compared to the control (1.24 nm), indicating minimal structural fluctuations. The radius of gyration (Rg) values also suggested that the complex remained compact, reinforcing its stability. Furthermore, Bayogenin formed an average of four hydrogen bonds with HK2, contributing to a strong and stable interaction. Targeting HK2 is crucial, as it plays a significant role in maintaining glycolysis in cancer cells. Our study suggests that Bayogenin may promote apoptosis by disrupting the HK2-VDAC complex, leading to cytochrome c release and activation of the mitochondrial apoptotic pathway. These findings indicate that Bayogenin could be a promising candidate for cancer therapy by interfering with key metabolic and apoptotic pathways [38].

Integrating Bayogenin, a compound derived from traditional medicinal plants, aligns with the need for culturally and geographically relevant therapeutic options. Its potential as a non-toxic, natural alternative highlights the importance of exploring phytochemicals for personalized medicine in resource-limited settings. By targeting pathways central to TNBC’s progression, Bayogenin could address the gaps in treatment accessibility and effectiveness, particularly in regions where resource-intensive therapies are less feasible.

PALB2 plays a crucial role in the repair of DNA double-strand breaks through homologous recombination, a critical repair mechanism. PALB2 interacts with both BRCA1 and BRCA2, forming complexes that facilitate the recruitment and stabilization of DNA repair factors at sites of damage. The PALB2-BRCA2 interaction is essential for the function of RAD51, a key protein involved in HR [39]. Studies have shown that mutations or loss of PALB2 can lead to defective DNA repair and increased sensitivity to DNA-damaging agents [40].

Bayogenin may act as a stabilizer of the PALB2-protein complexes, enhancing their function in DNA repair. By promoting the binding and stabilization of these complexes, bayogenin could facilitate the recruitment of DNA repair machinery, leading to more efficient repair of DSBs and reducing genomic instability. This enhancement of DNA repair could prevent the accumulation of mutations, thereby contributing to cell survival under stress conditions [41].

While the in-silico findings are promising, the computational nature of this study necessitates empirical validation through both in-vitro and in-vivo investigations. The pharmacokinetics and bioavailability of Bayogenin within clinical contexts also require further exploration to ensure its efficacy and safety. Future research should prioritize preclinical and clinical evaluations of Bayogenin’s therapeutic potential. Exploring its synergistic effects when combined with existing therapies, such as chemotherapy or targeted agents, may enhance its utility. Also, investigating Bayogenin’s broader impact on the molecular landscape of TNBC, including other apoptotic pathways and epigenetic modifications, is essential for fully understanding its therapeutic mechanisms.

## Conclusion

In conclusion, this study identifies Bayogenin as a novel phytochemical with strong binding affinity and stability toward key proteins involved in TNBC. Molecular dynamics simulations confirm its significant interactions with BRCA2, BAX, and PALB2, suggesting its potential role in modulating DNA repair and apoptosis pathways. These findings indicate that Bayogenin may serve as a promising natural alternative to existing treatments like Olaparib, though further experimental validation is required to confirm its therapeutic potential. This study is limited to computational analyses, and in vitro and in vivo experiments are necessary to validate these findings and assess Bayogenin’s clinical applicability.

## Electronic supplementary material

Below is the link to the electronic supplementary material.

Supplementary Material 1

## Data Availability

The authors confirm that the data supporting the findings of this study are available within the article and its supplementary materials.
