# Supplementary material for: Indian medicinal phytocompounds for targeting apoptosis and high-penetrance genes in triple-negative breast cancer: an in-silico exploration
Source: BMC Mol Cell Biol. 2025 Jul 30;26:24. doi: 10.1186/s12860-025-00548-6 (PMC12312594; doi:10.1186/s12860-025-00548-6)
Supplement: Supplementary file 1 — Supplementary Material 1 [file 12860_2025_548_MOESM1_ESM.docx]

**Phytochemicals Targeting Apoptosis and High-Penetrance Genes in Triple-Negative Breast Cancer: An In-Silico Exploration**

Reshmi Kumari and Satarupa Banerjee*

Department of Biotechnology, School of Biosciences and Technology, VIT University

Vellore, Tamil Nadu, India

**Tabel S1**

Swiss ADME parameter prediction for the top hits

| **Compound** | **Canonical Formula** | **MW** | **Rotatable** | **H-bond a** | **H-bond d** | **iLOGP** | **GI absorption** | **BBB permeant** | **Pgp substrate** | **Lipinski** | **violation** |
| --- | --- | --- | --- | --- | --- | --- | --- | --- | --- | --- | --- |
| Ajmaline | CCC1CC2C(C)C2H6N2 | 326.43 | 1 | 3 | 2 | 1.64 | High | Yes | No | Yes | 0 |
| alpha-Toxicarol | COC1cc2cC C23H22O7 | 410.42 | 2 | 7 | 1 | 3.8 | High | No | No | Yes | 0 |
| Bayogenin | OCC1(C)CC30H48O5 | 488.7 | 2 | 5 | 4 | 3.23 | High | No | Yes | Yes | 0 |
| Sym-homospermidine | NCCCCCNCC8H21N3 | 159.27 | 3 | 2 | 3 | 2.05 | High | No | No | Yes | 0 |
| Tephrosin | COC1cc2cC C23H22O7 | 410.42 | 2 | 7 | 1 | 3.79 | High | No | No | Yes | 0 |
| Olaparib | O=C(N1CC C24H23FN | 434.46 | 6 | 5 | 1 | 2.84 | High | No | Yes | Yes | 0 |

**Table S2**

Toxicity prediction for the top hits

| **Compounds** | **Carcinogenicity** | **Cytotoxicity** | **Immunotoxicity** | **Mutagenicity** |
| --- | --- | --- | --- | --- |
| Ajmaline | Non-toxic | Non-toxic | Non-toxic | Non-toxic |
| Bayogenin | Non-toxic | Non-toxic | Non-toxic | Non-toxic |
| Sym-homospermidine | Non-toxic | Non-toxic | Non-toxic | Non-toxic |
| Tephrosin | Non-toxic | Non-toxic | Non-toxic | Non-toxic |
| alpha-Toxicarol | Non-toxic | Non-toxic | Non-toxic | Non-toxic |
| Olaparib | Non-toxic | Non-toxic | Non-toxic | Non-toxic |

**Table S3**

Representation of the binding pocket of proteins, resolution, and grid box size around the binding site residues.

| **Protein Targets** | **PDB ID** | **Resolution (A⁰)** | **Ligand-binding pockets** | **Area (Å^2^) and Volume (Å^3)^** | **Number of residues** | **Binding Site Residue** | **Grid box** |
| --- | --- | --- | --- | --- | --- | --- | --- |
| BRCA1 | 1T15 |  | 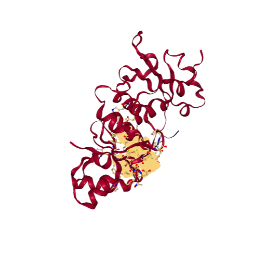 | 1110.81 and 733.18 |  | Val1654, Ser1655, Gly1656, Leu1657, Thr1658, Pro1659, Phe1662, Met1663, Thr1677, Asn1678, Leu1679, Ile1680, Met1689, Lys1690, Thr1691, Asp1692, Val1696, Cys1697, Glu1698, Thr1700, Leu1701, Lys1702, Leu1705, Val1740, Gln1779, Trp1782, Ser6, Thr7, Pro9, Thr10, Phe11 | 40×52×42 |
| BRCA2 | 3EU7 |  | 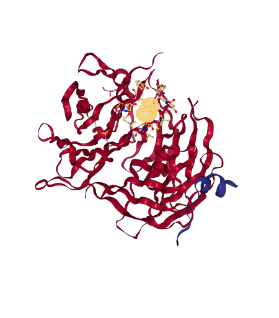 | 328.74 and 145.41 |  | Val872, Ser873, Ala874, Met875, Phe876, Ile888, Ile922, Val923, Pro924, Val925, Pro926, Asp927, Val928, Tyr929, Leu931, Val932, Cys933, Asp1122, Val1123, Lys1124, Lys1163, Trp1164, Ser1165, Gly1166 | 44×44 ×42 |
| TP53 | 4MZI |  | 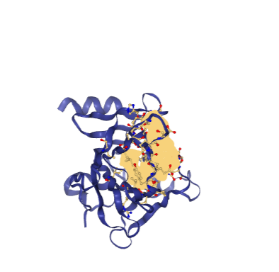 | 658.96 and 525.44 |  | Phe113, Leu114, His115, Se116, Ala119, Cys124, Thr125, Tyr126, Met133, Val141, Pro142, Gln144 | 56×48×55 |
| STK11 | 5WXN |  | 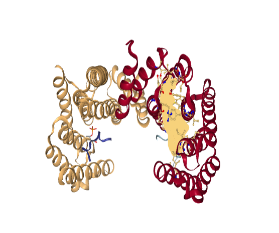 | 265.96 and 481.79 |  | Gln15, Ala16, Glu131, Arg8, Tyr19, Asp20, Asp21, Asn50, Val51, Ala54, Arg55, Ser58, Glu180, Glu89, Arg18, Tyr19, Asp20, Asp21, Asn50, Val51, Ala133, Arg333, Ser57, Ser58, Val61 | 48×48×54 |
| PTEN | 1D5R |  | 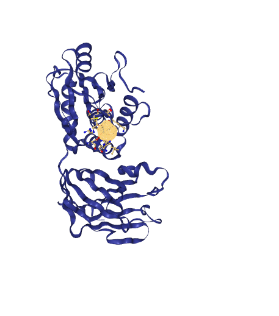 | 313.12 and 97.79 |  | Thr167, Pro169, Ser170, Ag72, Arg173, T yr176, Tyr177, Val275, Phe279, Ile280, Leu318, Leu320, Asn323, Asp324, Asn329, Lys330, Lys332 | 40×40 × 44 |
| PALB2 | 2W18 |  | 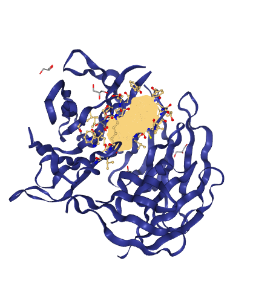 | 571.22 and 514.05 |  | Cys868,Ser869,Val870,Asp871,Ser873, Ala874, Met875, Phe876,Ile888,Glu892,Pro918,Val919,Leu920,Gln921,Ile922,Val923,Pro924,Val925,Pro926,Asp927,Val928,Tyr929,Leu931,Val928,Cys933,Gly937,Asn938,Leu939,Glu940,Ile941,Ile966,Lys967,Ala968,Val969,Leu970,Gly971,Thr973,Thr1012,Ile1013,Leu1014,Thr1015,Phe1016,Ala1017,Glu1018,Gln1020,Val1059,Cys1060,His1061,Lys1062,Ala1063,Phe1118, Leu1119, Glu1120, Gly1121,Asp1122,Val1123,Lys1124,Asp1125,Ile1131,Leu1132,Thr1133,Gly1135,His1158,Trp1159,Ser1160,Phe1161,Lys1163,Trp1164, Ser1165,Gly1166,Thr1167,Lys1176 | 44× 44×48 |
| BIRC5 | 2QFA |  | 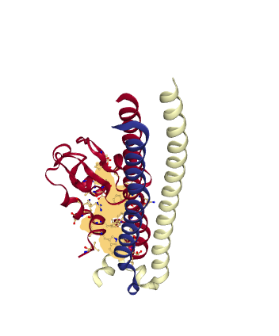 | 890.22 and 739.33 |  | Phe13, Leu14, Lys15, Asp16, Arg18, Arg37, Glu40, Ala41, Asp72, Pro73, Ile74, Phe86, Leu87, Ser88, Val89, Lys90, Lys91, Gln92, Phe93, Glu94 | 80×78×104 |
| BAX | 2G5B |  | 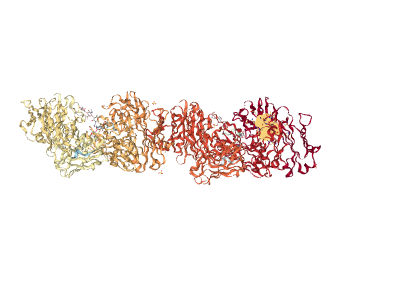 | 1028.87 and 651.83 |  | Ser9, Gln38, Lys39, Pro40, Gly41, Ala80, Glu81, Leu83, Ala84, Val85, Tyr87, Gly99, Gly100, Lys102, Leu103, Glu104, Ile105, Lys141, Trp162, Thr163, Asp164, Gln165, Asp166, Ser167, Lys168, Asp169, Ser170, Tyr172, Gln39, Gly42, Lys43, Ala44 | 68×98×104 |
| BCL2 | 6O0K |  | 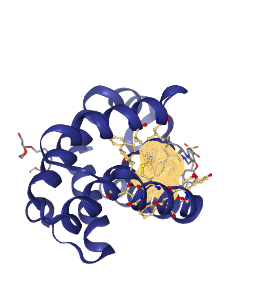 | 364.91 and 249.41 |  | Phe104, Ser105, Tyr108, Sp111, Phe112, Met115, Vl133, Leu137, L149, Glu152, Phe153, Vl156 | 62×50×56 |
| CASP3 | 1NME |  | 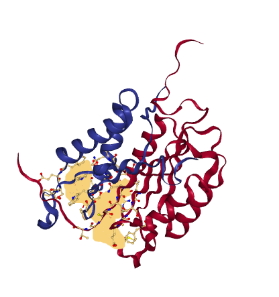 | 710.51 and 585.28 |  | Met61, Arg64, Ser65, Gly66, Thr67, Asp70, Leu119, Ser120, His121, Gly122, Glu123, Phe128, Gln161, Ala162, Cys163, Gly165, Thr166 | 88×62×84 |


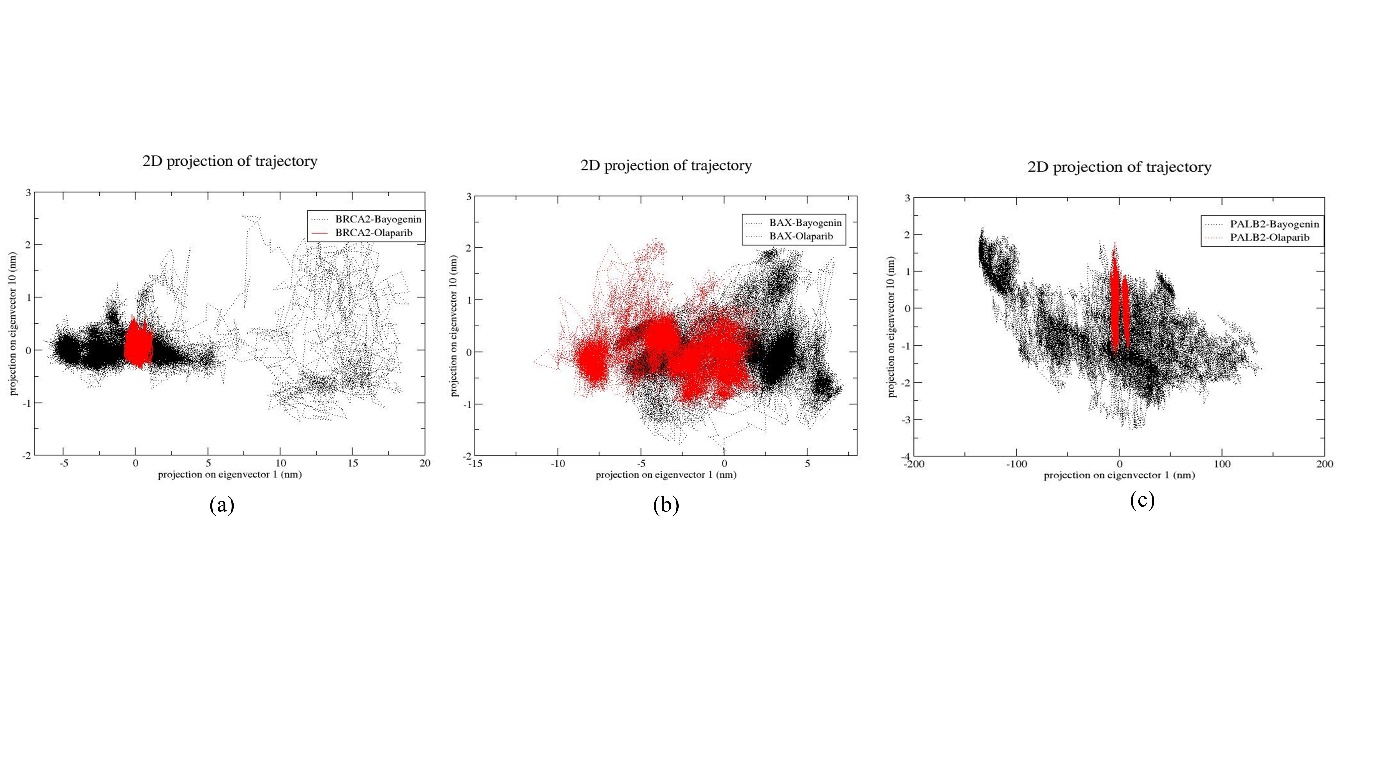


**Fig. S1.** Representation of PCA plot for (a) BRCA2-Bayogenin (black) and BRCA2-Olaparib (red), (b) BAX-Bayogenin (black) and BAX-Olaparib (red), (c) PALB2-Bayogenin (black) and PALB2-Olaparib (red) generated over 200 ns MD run.


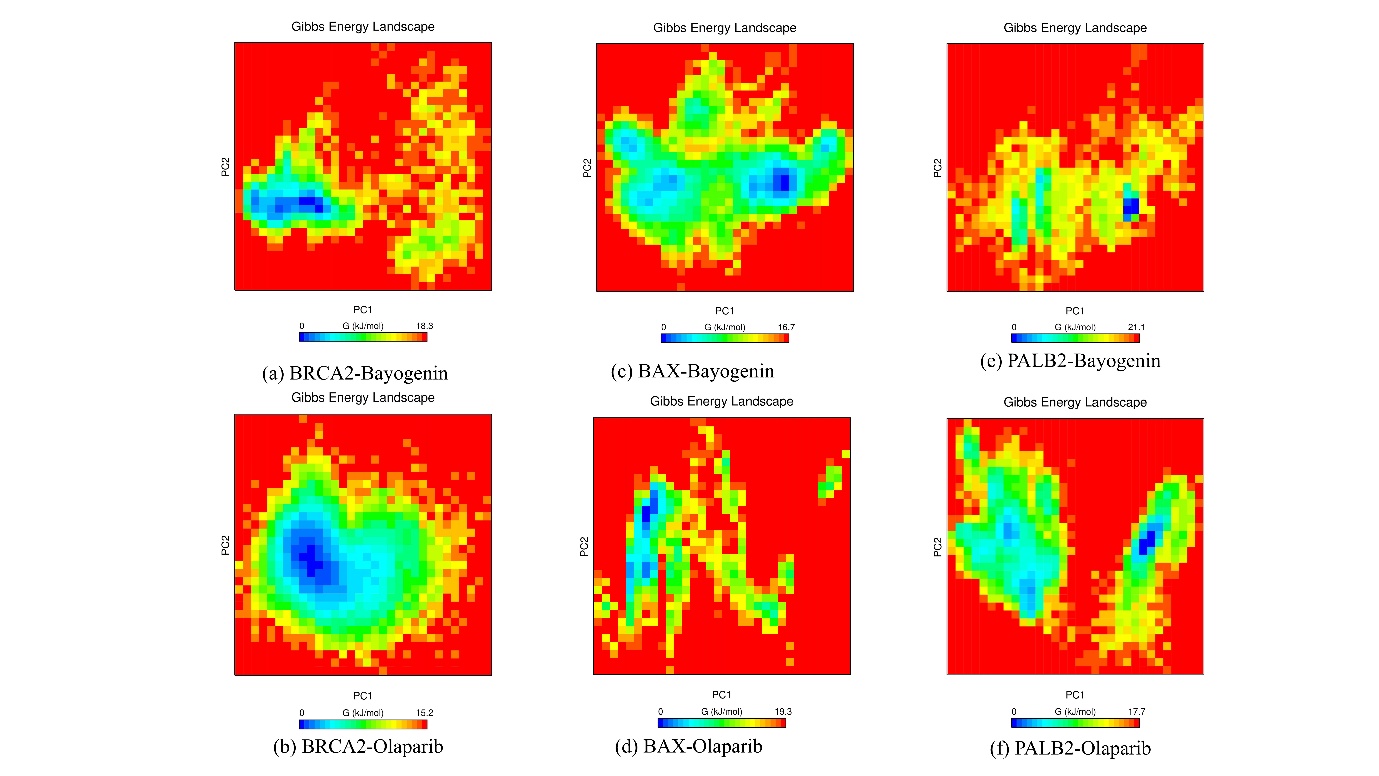


**Fig. S2.** FEL plot representation of (a) BRCA2-Bayogenin, (b) BRCA2-Olaparib, (c) BAX-Bayogenin, (d) BAX-Olaparib, (e) PALB2-Bayogenin, and (f) PALB2-Olaparib.
